# Mortality of apricot rootstocks and scions

**DOI:** 10.3389/fpls.2025.1707519

**Published:** 2026-02-11

**Authors:** Edina Mendelné Pászti, Géza Bujdosó, Gideon Sikwah Narteh, Zoltán Szabó, Ákos Mendel

**Affiliations:** Fruit Growing Research Center, Institute of Horticultural Sciences, Hungarian University of Agriculture and Life Sciences, Budapest, Hungary

**Keywords:** different rootstocks, Hungary, non-bearing period, survival rate, TCSA

## Abstract

Aim of our study is to assess the stability and profitability of apricot production by selecting some rootstock and scions among Hungarian climate conditions, that are good adapted to the changing climate. Comparative experiments will be conducted to gain comprehensive knowledge, including their impact on vegetative and generative development, viability, and mortality in new plantations. The experiment utilized a randomized block design, incorporating five rootstocks and 16 apricot scions, resulting in a total of 960 trees planted at a distance of 3 x 5 meters. The survival rate is expressed as a percentage of the initially planted trees for each combination. It can be concluded that rootstocks with vigorous growth, such as ‘Montcar’ and ‘Rootpac R’, were better suited to the climatic and soil conditions of the Hungarian lowlands compared to rootstocks with moderate growth potential, such as ‘Fehér besztercei’ and ‘Wavit’. This is due to their particularly vigorous root growth, which helps to maintain the plantation condition.

## Introduction

Grafting is a crucial technique in fruit tree cultivation, involving the fusion of two genetically distinct individuals: the rootstock and the scion. This process promotes a strong symbiotic relationship between the two, resulting in optimal growth and development (Gündoğdu, 2019; Shahkoomahally et al., 2020). Rootstocks have a significant impact on tree physiology and productivity, including vegetative growth, resistance to soil pathogens and nematodes, flowering behavior, fruit yield, and quality (Beckman et al., 1992; Errea et al., 1994; Layne, 1994; Boyhan et al., 1995). As a result, growers often search for new cultivars that provide better fruit characteristics, longer post-harvest life, and higher overall yield. However, it is essential to assess the climatic adaptability of these cultivars in addition to their agronomic traits (Bassi and Audergon, 2006; Piagnani et al., 2013).

Apricot seedlings are the most commonly used rootstocks for apricot cultivation in Central and Eastern Europe, as well as in Asia. Significant progress has been made through the generative breeding of Hungarian rootstocks, specifically the C.1301, C1650, and C.1652 varieties, which were carefully selected in Cegléd during the late 1980s (Pászti and Mendel, 2018). Usage of apricot seedlings as rootstocks has become popular in the mentioned regions due to their compatibility and performance with various apricot cultivars (Rom, 1989). Hungarian rootstocks, which were developed through systematic breeding efforts, have shown great promise in terms of adaptability and compatibility. The selection process, which took place in Cegléd (Hungary) during the late 1980s, highlights the significance of targeted breeding programs in improving the performance and resilience of fruit tree rootstocks. However, despite the progress made in rootstock development, challenges remain regarding the adaptability of certain cultivars and rootstocks to varying climatic condition (Mendelné Pászti et al., 2022).

These particular rootstocks have shown excellent compatibility with a variety of apricot cultivars (Hernández et al., 2010). However, it is crucial to conduct thorough evaluations of the climatic suitability of different rootstock options, especially those associated with apricot, cherry, and walnut species, due to the inherent limitations in adaptability observed in certain cultivars and rootstocks (Dos Santos Pereira et al., 2014; Bujdosó et al., 2019). When selecting rootstocks and cultivars for fruit tree cultivation, it is crucial for growers and researchers to prioritize the assessment of climatic adaptation alongside other agronomic traits. The dynamic nature of environmental conditions requires a proactive approach to ensure the sustainability and productivity of orchard systems. Ongoing research efforts to enhance the genetic diversity and resilience of fruit tree populations will play a crucial role in addressing emerging challenges and opportunities in fruit tree cultivation (Chokoeva and Marinov, 1997). Collaborative endeavors and strategic investments in research and development can help the fruit industry thrive while mitigating the impacts of environmental variability and changing climatic conditions. The accuracy and reliability of the analyses performed rely on the availability of dependable plant materials (Mendelné Pászti and Mendel, 2021).

The primary aim of this study was to evaluate the suitability of selected rootstocks and scion cultivars for long-term orchard establishment under managed field conditions and to determine whether there is a correlation between tree survival and basic growth parameters.

## Materials and methods

The experiment took place at the Cegléd Research Station, located at the Fruit Growing Research Centre of the Hungarian University of Agriculture and Life Sciences. The region has a temperate, continental climate with a semi-arid microclimate, and an altitude of 96 meters above sea level.

The irrigated orchard was planted during spring of 2018 with a spacing of 3 x 5 m. The trial was established on chernozem soil characterized by a high lime content (pH 7.24; total CaCO_3_ in the upper 30 cm: 7.25%) and an elevated humus level (3.27%). The soil properties of the orchard are summarized in **Table 1**. According to the Arany-type cohesion index, a Ka value of 40.27 indicates medium soil compactness.

**Table 1:** Some physical and chemical properties of the research area soil.

Soil properties	Value	Limits	Evaluation
pH (KCl)	7,24	0-14	Slightly alkaline
Organic matter (%)	3,27	1-5	Medium
Total saline (%)	0,03	0,01-0,50	Non-saline
CaCO_3_ (%)	7,25	1-20	High
Available nitrogen (mg*kg^-1^)	5,06	1-10	Medium
Available phosphorous-pentoxide (mg*kg^-1^)	197,13	0-350	Excellent
Available potassium-oxide (mg*kg^-1^)	257,87	0-350	Medium
Available sodium (mg*kg^-1^)	70,93	0-100	Medium
Available magnesium (mg*kg^-1^)	246,60	40-300	Good
Available sulphate sulphur (mg*kg^-1^)	15,68	4-35	Medium
Available manganese (mg*kg^-1^)	36,00	10-50	Good
Available zinc (mg*kg^-1^)	1,50	0,7-3	Good
Available copper (mg*kg^-1^)	4,11	0-6	Good
KA	40,27	1-100	Medium

The data collected during the third growing season’s autumn (2021) are analyzed.

‘Fehér besztercei’ is a vegetatively propagated apricot rootstock that is highly compatible with Hungarian apricot cultivars. Due to the long lifespan of the trees grafted on ‘Fehér besztercei’, this rootstock is recommended for use in Hungary. During the past decade, ‘Myrobalan 29C’ has been increasingly used in the Mediterranean region, due to its tolerance for compact and poorly aerated soils. ‘Wavit’ was chosen for its low growth vigor in high density systems. ‘Montclar’ and ‘Rootpac R’ are rootstocks with strong roots, making them ideal for replanting or for use on poor soils (**Table 2**).

**Table 2:** Used rootstock cultivars and their species.

Rootstocks	Species
‘Fehér besztercei’ (Fb)	*Prunus domestica* L.
‘Montclar’ (MC)	*Prunus persica* L.
‘Myrobalan’ 29C (My)	*Prunus cerasifera myrobalana* Ehrh.
‘Rootpack R’ (RR)	*P. cerasifera myr.* X *P. dulcis* Mill.
‘Wavit’ (Wv)	*Prunus domestica* L.

The micropropagated rootstocks were established in the outdoor nursery in spring as plug-grown transplants. Budding with the selected scion cultivars was carried out in August using dormant (T-)budding techniques. The bare-root engraftments were ready for orchard establishment in the following autumn.

The scions include traditional cultivars from Hungary such as ‘Ceglédi óriás’, ‘Ceglédi szilárd’, ‘Gönci magyar kajszi’, and ‘Pannónia’, as well as cultivars from the USA like ‘Flavor Cot’, ‘Goldrich’, ‘Lilly Cot’, ‘Spring Blush’ and ‘Tom Cot’), France (including ‘Bergeron’, ‘Bergarouge’, ‘Lady Cot’, ‘Pink Cot’, and ‘Tardif de Valence’), Canada (‘Harogem’), and Afghanistan (‘Roxana’).

In grafted fruit trees, mortality may arise from various biotic and abiotic factors, including pests, pathogens, water stress, mechanical injuries, or inadequate orchard management. In our case, the orchard was irrigated and regularly supplied with nutrients and pruning; rootstock–scion incompatibility was not observed, and no mechanical damage occurred. The site had been under arable cultivation (cereal and row crops in rotation) for the preceding thirty years, and plant protection was applied according to regular monitoring. Therefore, the observed tree losses may have originated from soil-borne pathogens (e.g., *Verticillium* spp.), bacterial infections (e.g., *Pseudomonas* spp.), or phytoplasma infections (‘*Candidatus Phytoplasma prunorum*’).

As the primary aim of the study was not to assess the susceptibility of the rootstocks or scion cultivars to the possible infections, but rather to evaluate their general suitability for long-term orchard establishment, the cause of death of individual trees was not classified according to these potential factors.

Furthermore, the trunk diameter was measured at 20 cm above graft union in November 2021 to calculate the trunk cross sectional area (TCSA). The tree height of the rootstock/scion combinations was measured also at the end of the vegetation season.

The data is analyzed using one-way univariate analysis of variance (ANOVA). The statistical procedures were conducted using IBM SPSS v.27 software. **Table 3** summarizes the survival rate (SR) of the rootstock-scion combinations.

**Table 3:** Survival rate (in %) of scion-rootstock combinations, in 3^rd^ leaf.

	Bergarouge	Bergeron	C. óriás	C. szilárd	Flavor Cot	Goldrich	Gönci	Harogem	
Fb	83	58	80	75	67	0	83	100	
Mc	94	92	92	83	89	92	81	83	
My	62	73	92	92	83	73	94	100	
RR	92	100	100	92	92	83	100	92	
As	80	67	56	33	58	0	80	89	
Wv	50	100	0	100	67	44	73	100	
Mean	77	82	70	79	76	49	85	94	

	Lady Cot	Lily Cot	Roxana	Spring Blush	Tardif de V.	Tom Cot	Pannónia	Pink Cot	Mean
Fb	92	75	11	89	92	83	100	33	68
Mc	100	92	92	100	92	100	100	92	88
My	81	67	92	67	92	44	83	100	84
RR	100	83	75	83	100	100	83	75	94
As	100	92	47	0	75	33	0	78	58
Wv	83	0	0	0	50	100	50	83	67
Mean	93	68	53	56	83	77	69	77	76

## Results and discussion

The study utilizes the survival rate (SR) expressed as a percentage of the originally planted trees for each combination (**Table 3**).

Analysis of the data collected in the third leaf of the experimental orchard revealed significant insights into the compatibility between different grafting partners. It was evident that there were certain incompatibilities among some combinations. Notably, the combination of ‘Wavit’ (comprising ‘C. óriás’, ‘Lilly Cot’, ‘Roxana’, and ‘Spring Blush’) had the lowest survival rate (SR) of 0%. Conversely, some cultivars such as ‘Bergeron’, ‘C. szilárd’, ‘Harogem’, and ‘Tom Cot’ achieved remarkably high survival rates of 100%. It is interesting to note that combinations involving ‘Goldrich’, ‘Spring Blush’, and ‘Pannonia’ standing on yielded ‘Apricot seedling’ a 0% survival rate. Additionally, ‘Fehér besztercei’ exhibited a 0% survival rate only when ‘Goldrich’ grafted on it. Conversely, the combinations Fb (‘Harogem’, ‘Pannonia’), Mc (‘Lady Cot’, ‘Spring Blush’, ‘Tom Cot’, and ‘Pannonia’), My (‘Harogem’, ‘Pink Cot’), RR (‘Bergeron’, ‘C. óriás’, ‘Gönci m.k.’, ‘Lady Cot’, ‘Tardif de V.’, and ‘Tom Cot’), and ‘As’ (‘Pink Cot’) all displayed a 100% survival rate.

The survival rates (SR) for the rootstocks were highest on RR (94%) combinations, followed closely by Mc (88%), My (84%), Fb (68%), and Wv (67%). Notably, ‘Myrobalan 29C’ demonstrates commendable adaptability, especially in soils with high lime content (Foschi et al., 2012). However, the usefulness of myrobalan has been controversial for many years due to the physiological incompatibility between scion and rootstock trees and tree mortality (Grzyb et al., 1998; Sosna and Lichnar-Malanczuk, 2012). Although less utilized in Hungary (Bakos et al., 2022), the RR and Mc rootstocks exhibited the most robust survival rates in this experiment.

The ranking of the SR values for individual cultivars further clarifies the variations in graft compatibility. Mortality was also influenced by cultivar, as reported by Sosna and Licznar-Małańczuk (2012). Notably, ‘Harogem’ was the top performer with a remarkable 94% survival rate, closely followed by ‘Lady Cot’ (93%), ‘Gönci m. k.’ (85%), ‘Tardif de V.’ (83%), ‘Bergeron’ (82%), and ‘C.’ ‘Szilárd’ had the highest survival rate at 79%, followed by ‘Bergarouge’, ‘Tom Cot’, and ‘Pink Cot’ at 77% each, ‘Flavor Cot’ at 76%, ‘C. óriás’ at 70%, ‘Pannonia’ at 69%, ‘Lilly Cot’ at 68%, ‘Spring Blush’ at 56%, ‘Roxana’ at 53%, and ‘Goldrich’ at 49%. The average survival rate in the third leaf across the entire experimental orchard was 76%.

All six rootstocks had a significant impact on the survival rate, with the scion also having a moderate influence. The good survival rate of the ‘Bergeron’ variety and the high mortality rate of the ‘Goldrich’ variety were already known from previous experiments (Licznar-Małańczuk and Sosna, 2005; Sosna and Licznar-Małańczuk, 2012). The data suggests that robust rootstocks like Mc and RR were better suited to the climatic and soil conditions of the Hungarian lowlands compared to rootstocks with moderate growth potential such as Fb and Wv. The robust root growth associated with vigorous rootstocks significantly contributed to the overall health and vigor of the orchard plantation. This phenomenon had already been highlighted by Egea et al. (1989). In addition, some scions, such as ‘Harogem’, ‘Lady Cot’, ‘Gönci m.k.’, ‘Tardif de V.’, and ‘Bergeron’, showed better compatibility with the tested rootstocks than others, such as ‘Spring Blush’, ‘Roxana’, and ‘Goldrich’. This emphasizes the significance of carefully selecting rootstock-scion combinations to enhance graft compatibility and overall orchard performance.

Our results demonstrate that the tested rootstocks and scion cultivars were generally suitable for long-term orchard establishment under managed field conditions, with survival rates and basic growth parameters showing promising performance. This aligns with previous studies showing that the propagation method, whether micropropagated or from cuttings, does not necessarily affect field performance when standard orchard management is applied (Lezzer et al., 2022; Marín et al., 2023). Rootstock–scion interactions remain a critical determinant of tree growth and survival. Long-term apple and pear trials have highlighted that specific rootstock × scion combinations significantly influence trunk cross-sectional area, leaf nutrient status, and yield efficiency, confirming that compatible combinations are essential for sustained orchard performance (Valverdi and Kalcsits, 2021; Shuttleworth et al., 2023). Our study’s focus on survival and growth parameters rather than pathogen susceptibility is consistent with these findings, emphasizing practical considerations for orchard establishment.

Soil conditions and rootstock adaptation also play an important role. Research on fruit trees in heavy-calcareous soils has shown that rootstock choice can significantly influence mineral uptake, growth, and overall tree performance (Xu and Liu, 2021; Xu et al., 2021). In our trial, the history of arable cultivation may similarly have affected growth variation among trees, highlighting the need for rootstocks adapted to local soil conditions. Mechanistic studies of graft union formation and rootstock–scion physiological interactions further support our findings. Vascular reconnection, callus formation, and biochemical signaling at the graft interface are essential for graft success and long-term survival (Jain et al., 2025; Kviklys and Samuolienė, 2020). While these processes were not directly studied in our trial, the high survival and uniform growth indicate that they proceeded effectively across the tested combinations.

Finally, the system-level nature of grafted plants, in which scion genotype can influence rootstock function and nutrient uptake, reinforces the importance of evaluating survival together with growth parameters under realistic orchard conditions (Xu et al., 2022; McLean et al., 2025; Albrecht and Syvertsen, 2017). Our study contributes to this body of knowledge by providing practical evidence that the tested rootstocks are suitable for long-term orchard management, supporting informed decisions for growers and highlighting the value of field-based evaluation alongside physiological and molecular insights.

For further investigations, regression analyses were performed with the vegetative characteristics. Five vegetatively propagated rootstocks were included in the regression analysis of tree height and survival rate. The rootstocks were ‘Montclar’ (Mc), ‘Myrobalan 29C’ (My), ‘Wavit’ (Wv), ‘Rootpac R’ (RR) and ‘White Besztercei’ (Fb). Total height of trees (TH), trunk cross-sectional area (TCSA) and canopy volume (CV) were the dependent variable, with survival rate as the independent variable. Data from 2021 were selected because the trees reached the desired height in the plantation that year. The regression analysis included linear, inverse, and exponential functions. A total of 549 individuals were analyzed, with the values averaged per subject. The normality of the error terms was accepted based on the Shapiro–Wilk test (TH: K(5)= 0.97; p = 0.85; TCSA: K(5)=0,88;p=0,30; CV: K(5)=0,98;p=0,93).

**Figure 1:**
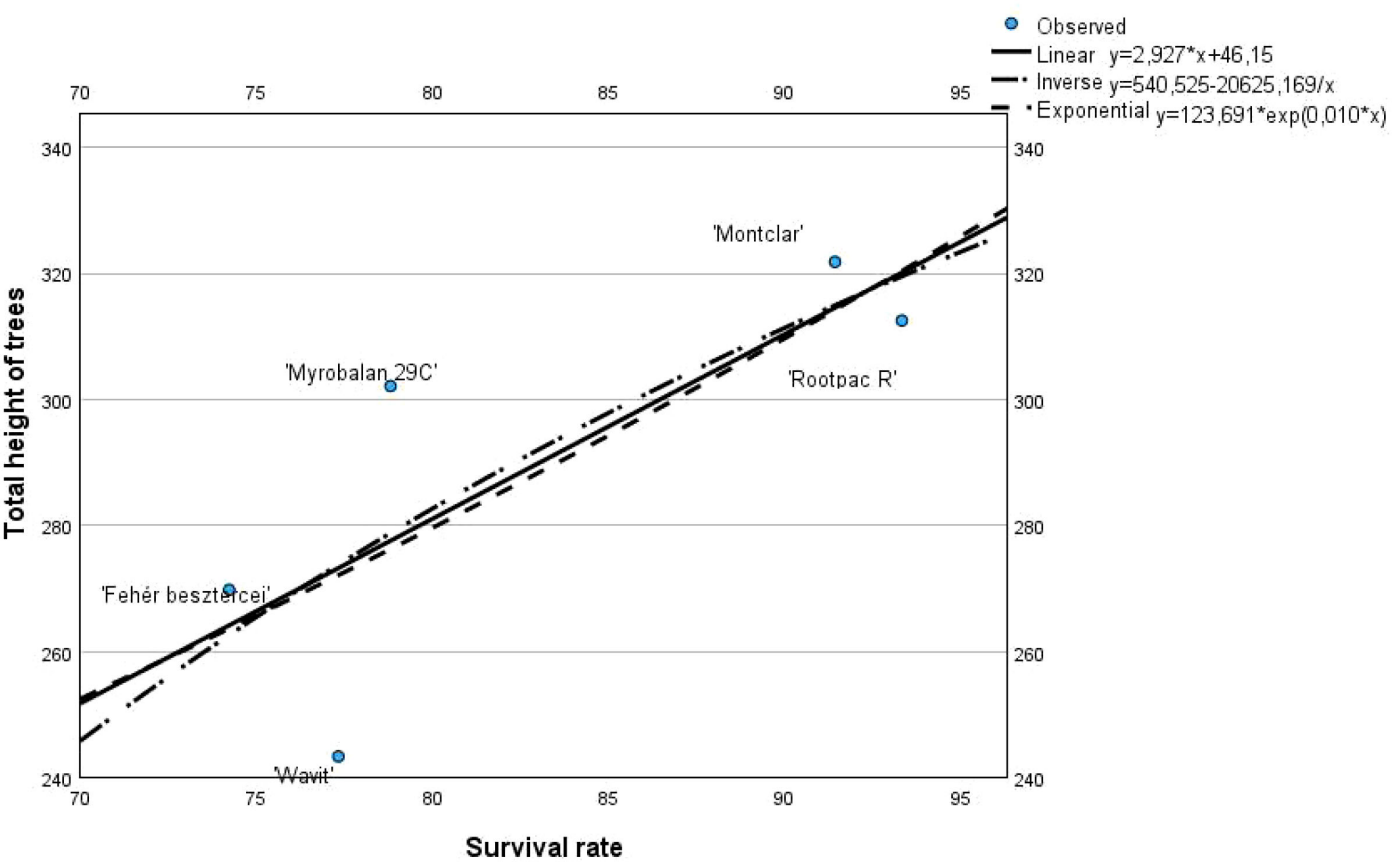
Correlation between the tree height and the survival rate in the third leaf (Cegléd, 2021).

The R² values are obtained from the regression diagnostics, F-test and t-test for the three different functions show that the values are very similar and can be interpreted and accepted. Based on these values, the inverse model is the best fit, with R² = 0.619, F(1, 3) = 4.88, p = 0.114 and t = -2.21 (**Figure 1**).

There is a clear positive correlation between survival rate and tree height: as the survival rate increases, the total height of the trees tends to increase as well. This is evident from all three fitted models, which show a rising trend. The linear regression model, described by the equation y = 2.927x + 46.15, suggests a consistent, proportional increase in tree height with each percentage point increase in survival rate. The exponential model (y = 123.691 × exp(0.010 × x)) implies a slightly accelerating growth in height at higher survival rates, while the inverse model (y = 540,525 - 20625.169/x) suggests diminishing returns—meaning the benefit in height gained from increases in survival rate becomes less significant as survival approaches the higher range.

When looking at individual cultivars, ‘Montclar’ and ‘Rootpac R’ stand out with both high survival rates (above 90%) and tall tree heights (above 320 cm), placing them above or near all regression lines. These cultivars demonstrate strong overall performance. In contrast, ‘Wavit’ exhibits the lowest values for both survival and height, indicating weaker adaptability or vigor under the tested conditions. ‘Myrobalan 29C’ presents an interesting case: despite a moderate survival rate (~79%), it achieves a higher-than-expected height (~300 cm), suggesting potential advantages in growth even if survivability is not optimal. Meanwhile, ‘Fehér besztercei’ shows both lower survival and moderate height, placing it below the linear trend.

In summary, the chart emphasizes a strong and consistent relationship between tree survival and growth, validated by the similarity among the regression models. The data suggest that cultivars with higher survival rates generally develop greater total height, making survival rate a reliable indicator of overall tree performance. This analysis can help in selecting the most suitable rootstocks for cultivation based on both survival and growth characteristics.

**Figure 2:**
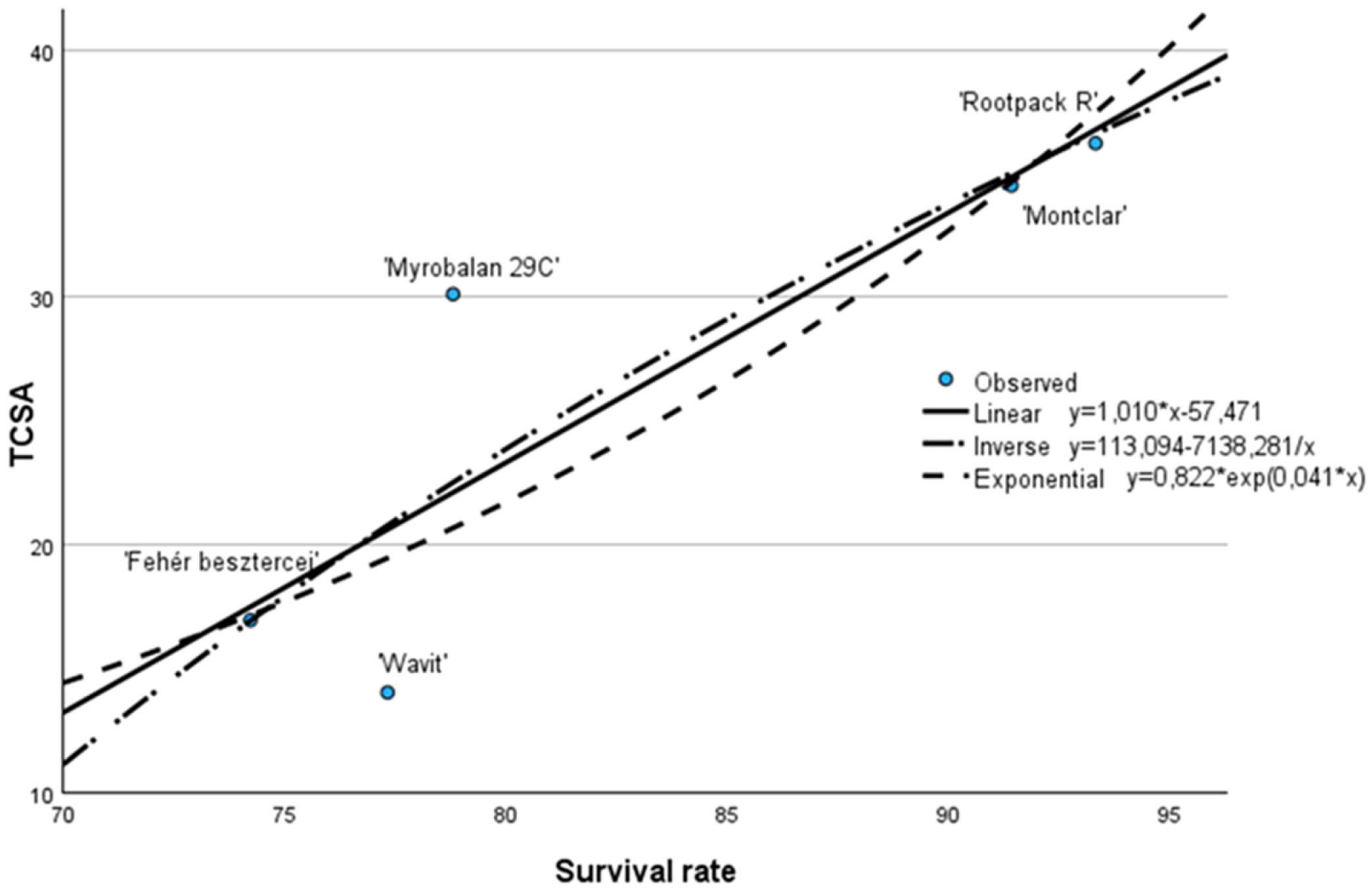
Correlation between the TCSA and the survival rate in the third leaf (Cegléd, 2021).

As in the previous case, the R² values for the three different functions tested in the regression diagnostics, the F-test and the t-test showed very similar results. Based on the values obtained, the inverse model provides the best fit, with R² = 0.752, F(1, 3) = 9.09, p = 0.057 and t = -30.1 (**Figure 2**).

Similar to the previous chart, the results show a positive correlation between survival rate and TCSA, meaning that trees with higher survival rates tend to develop thicker trunks. The linear regression equation (y = 1.010x - 57.471) suggests that for every 1% increase in survival rate, TCSA increases by approximately 1.01 cm². This linear trend aligns well with the distribution of the observed data.

The exponential model (y = 0.822 × exp(0.041x)) indicates a slightly accelerating growth in TCSA as survival rate increases. In contrast, the inverse model (y = 113.094 - 7138.281/x) implies a steep rise in TCSA at lower survival rates, followed by a plateau at higher survival rates. All three models display a similar general trend, reinforcing the reliability of the observed correlation.

Examining the individual cultivars, ‘Rootpac R’ and ‘Montclar’ again demonstrate the best performance, with high survival rates (~90–94%) and TCSA values over 35 cm², placing them well above the trend lines. These cultivars are likely the most vigorous in terms of both survival and trunk development. On the other hand, ‘Wavit’ shows both low survival and the lowest TCSA (~14 cm²), confirming its weaker growth potential. Interestingly, ‘Myrobalan 29C’ stands out by having a moderate survival rate (~79%) but a relatively high TCSA (~31 cm²), indicating strong growth despite somewhat lower survival. ‘Fehér besztercei’ shows relatively low values in both categories, with TCSA around 19 cm².

In conclusion, the chart confirms a strong positive relationship between survival rate and trunk development across the studied cultivars. The consistent alignment of the data with all three regression models underlines the robustness of the trend. Cultivars like ‘Rootpac R’ and ‘Montclar’ emerge as clear leaders, combining strong survivability with vigorous growth, while ‘Wavit’ appears least favorable under the examined conditions.

In this case, we also tested the R^2^ estimates of three different functions. We also tested the F-test and the t-test. The results show that the obtained values are very similar. They are also acceptable. As was the case previously, the inverse model is the one that best fits the data, with an R^2^ of 0.767, an F(1,3)=9.85; p=0.052, t:-3.14.

These findings highlight the complex nature of graft compatibility, as some combinations show good compatibility while others have limited survival. It is important to carefully select cultivars and rootstocks in orchard management practices. Understanding these nuances is crucial for orchard management and cultivar selection, as it enables growers to make informed decisions to maximize productivity and yield. Further research is necessary to enhance our understanding of the underlying mechanisms governing graft compatibility. This will facilitate more effective orchard management practices.

## Data Availability

The datasets presented in this study can be found in online repositories. The names of the repository/repositories and accession number(s) can be found in the article/supplementary material.
